# Health Insurance as a Mediator of Neighborhood Deprivation and Pediatric Cancer Survival: An Analysis of State Cancer Registry Data

**DOI:** 10.1002/cam4.71755

**Published:** 2026-03-27

**Authors:** Emma Hymel, Cheng Zheng, Jenna Allison, Kendra L. Ratnapradipa, Edward S. Peters, Sarah H. Nash, Mei‐Chin Hsieh, Shinobu Watanabe‐Galloway

**Affiliations:** ^1^ Department of Epidemiology, College of Public Health University of Nebraska Medical Center Omaha Nebraska USA; ^2^ Department of Biostatistics, College of Public Health University of Nebraska Medical Center Omaha Nebraska USA; ^3^ Department of Pediatrics, College of Medicine University of Nebraska Medical Center Omaha Nebraska USA; ^4^ Iowa Cancer Registry, Department of Epidemiology, College of Public Health University of Iowa Iowa City Iowa USA; ^5^ Department of Epidemiology and Population Health, School of Public Health LSU Health Sciences Center New Orleans Louisiana USA

**Keywords:** cancer, insurance, pediatric, survival

## Abstract

**Introduction:**

To develop interventions to reduce neighborhood‐level disparities in pediatric cancer outcomes, it is necessary to understand their underlying mechanisms. It has been suggested that individual‐level health insurance is a mediator of neighborhood deprivation and pediatric cancer survival.

**Methods:**

This study was a population‐based longitudinal study of children with cancer from 2000 to 2020 in the Iowa Cancer Registry and Louisiana Tumor Registry. Neighborhood deprivation was measured using the Area Deprivation Index. Log‐binomial regression models were used to identify predictors of health insurance status at diagnosis. Cox regression models were used to assess the association between health insurance status at diagnosis and cancer‐specific survival. Causal mediation analyses were conducted to investigate whether health insurance status serves as a mediator of the relationship between neighborhood deprivation and survival.

**Results:**

The study included 5782 children with cancer: 2069 in Iowa and 3723 in Louisiana. Children in more deprived neighborhoods, non‐White children, and children in Louisiana were more likely to have non‐private insurance. Compared with children with private insurance, those with non‐private insurance had a 32% higher hazard of cancer death (aHR = 1.32, 95% CI: 1.13–1.55). Insurance status was observed to mediate the association between ADI and cancer‐specific survival, mediating 7.33%–14.59% of the estimated association.

**Conclusion:**

While individual‐level health insurance status was a mediator of neighborhood‐level disparities in pediatric cancer survival, it did not explain a large proportion of the observed disparities. This suggests that structural and systemic factors, beyond just individual insurance coverage, may play a significant role in shaping pediatric cancer outcomes.

## 1 Introduction

Every year, approximately 400,000 children (individuals aged 0–19 years) are diagnosed with cancer globally, with about 15,000 of those cases occurring in the United States [1, 2]. Incidence of pediatric cancer has generally been increasing in the United States over the last two decades from 164.5 cases per million in 2003 to 177.2 cases per million in 2019 [3], and in 2024, it was estimated that 1590 children would die from cancer in the United States [4]. Social drivers of health (SDOH), or the conditions in which we live, work, learn, and play, are fundamental causes of health outcomes, including cancer incidence and mortality [5]. Despite advances in treatment and clinical trials, SDOH‐related disparities in pediatric cancer outcomes persist [6]. For example, children living in areas characterized by high levels of neighborhood deprivation as measured by the Area Deprivation Index (ADI), a composite measure of 17 indicators of education, employment, income, and housing, have an increased hazard of death compared with children living in less deprived areas [7, 8].

There is a growing interest in developing interventions to mitigate pediatric cancer disparities [6, 9]. For example, a systematic approach to addressing SDOH known as social prescribing, in which providers prescribe non‐clinical activities, can be integrated into patient care [10]. To develop these interventions, it is necessary to understand the causal pathways linking SDOH with cancer outcomes. Causal mediation analysis can be used to investigate these underlying mechanisms, as it seeks to determine causal relationships between exposure and outcome, estimating both the direct effect of the exposure on the outcome and indirect/mediated effects on the outcome through a mediator variable [11].

Health insurance may act as a mediator between neighborhood deprivation and pediatric cancer survival [7]. In two cancer registry‐based studies, children, adolescents, and young adults with no health insurance and those covered by Medicaid experienced lower survival, even after adjusting for cancer stage [12, 13]. In a study of Surveillance, Epidemiology, and End Results (SEER)‐Medicaid linked data, Medicaid enrollment at any point six months before or six months after diagnosis was associated with increased odds of later stage at diagnosis and increased hazard of death among children with cancer [14]. Further, in an analysis of the National Cancer Database, health insurance type was a mediator of racial/ethnic pediatric cancer survival disparities [15]. While a lower proportion of children are uninsured (5%) compared with adults (12%), approximately 39% of children have public insurance (i.e., Medicaid, Children's Health Insurance Program), with higher rates of public insurance among children in low‐income families and children with special health needs [16].

It is not yet clear whether health insurance‐based disparities in pediatric cancer outcomes are due to the differences in insurance type, or if the disparities are fundamentally caused by socioeconomic deprivation, of which insurance status serves as a proxy [17]. To date, no studies have performed a causal mediation analysis to identify mediators of the association between ADI and pediatric cancer survival. Thus, the objectives of this study were to identify (1) whether neighborhood deprivation is associated with health insurance, (2) whether health insurance is associated with pediatric cancer survival, and (3) whether health insurance is a mediator of the association between neighborhood deprivation and survival.

## 2 Methods

### 2.1 Study Population

This population‐based longitudinal study used data from the Iowa Cancer Registry and Louisiana Tumor Registry. The study was approved by the Institutional Review Board at the University of Nebraska Medical Center (0367‐23‐EX), and was approved by the University of Iowa and Louisiana State University Health Sciences Center. The study cohort consisted of individuals with a primary diagnosis of cancer at ages 0 to 19 in Iowa and Louisiana from 2000 to 2020. Cases diagnosed only by autopsy or death records were excluded by the cancer registries. Data from each state were combined before applying the additional exclusion criteria. Children diagnosed with non‐malignant tumors or tumors in situ were excluded. Health insurance status was not a required data item in Iowa before 2007, so those cases missing health insurance information and any other cases missing data on health insurance from either state were excluded from the analysis.

### 2.2 Variables

#### 2.2.1 Survival Time

Survival time was measured in months as the time from diagnosis to death or last follow‐up using vital status through December 31, 2020. Vital status and cause of death are ascertained through annual linkage to the National Death Index, as well as local death clearance, which includes death certificate review. The primary outcome of interest was cancer‐specific mortality.

#### 2.2.2 ADI

Patient addresses at diagnosis were geocoded by each cancer registry. The main exposure of interest was the ADI. National ADI rankings were obtained from the Neighborhood Atlas, Version 3, corresponding to census block groups [18]. The values of the ADI can change depending on the reference population; national rankings were used instead of state‐specific rankings to allow for comparison across states. The ADI is derived from a five‐year average of American Community Survey (ACS) data, utilizing established methodologies [19, 20]. ADI rank quartiles were categorized in the analyses as follows: Q1 (1–25), Q2 (26–50), Q3 (51–75), and Q4 (76–100), consistent with previous research [8, 21]. Q1 is the least deprived and Q4 is the most deprived. For cases diagnosed between 2000 and 2010, ACS data from 2005 to 2009 were used; for those diagnosed between 2011 and 2020, ACS data from 2013 to 2017 were used.

#### 2.2.3 Health Insurance

Health insurance status at the time of diagnosis was abstracted from the registry data as the primary payer at diagnosis. Insurance was categorized as private, public, insured but not otherwise specified (insured NOS), or uninsured. A binary version of health insurance (private vs. non‐private insurance) was also created; those who had public insurance, insured NOS, or uninsured were considered to have non‐private insurance.

#### 2.2.4 Other Variables

Race and ethnicity were combined and categorized as non‐Hispanic White, non‐Hispanic Black, non‐Hispanic other, or Hispanic (any race). Cancer type was classified according to the International Classification of Childhood Cancer, Third edition (ICCC‐3) codes. Cancers were grouped as hematologic malignancies (ICCC groups 1 and 2), central nervous system (CNS) tumors (ICCC group 3), and other extracranial solid tumors (ICCC groups 4–12). Rurality was measured using 2010 Rural–Urban Commuting Area (RUCA) codes defined by the US Department of Agriculture at the Census tract level as urban (RUCA codes 1–3) or rural (RUCA codes 4–10) [22]. Additional variables considered included sex, age at primary diagnosis, and year of diagnosis.

### 2.3 Statistical Analysis

All analyses were conducted in R (version 4.4.0). To handle missing data on race/ethnicity (0.77%), rurality (0.13%), survival time (0.29%), ADI (10.31%), and cause of death (0.44%), the missing data were multiply imputed five times using the mice package in R. Descriptive statistics of the imputed data were computed and chi‐square tests and *t*‐tests were used to test for group differences by state, as appropriate. Additionally, to examine changes in health insurance before and after the implementation of the Patient Protection and Affordable Care Act (ACA), we compared insurance status before and after implementation. While ACA was signed into law in 2010, Medicaid expansion was not fully implemented until 2014 in Iowa and 2016 in Louisiana. The post‐ACA period was defined as 2014–2020 in Iowa and 2016–2020 in Louisiana [23, 24].

A directed acyclic graph (DAG) was used to identify the minimal sufficient adjustment set needed to control for confounding (Figure S1) [25]. Log‐binomial models were used to estimate the prevalence ratios (PR) for the association between ADI and binary health insurance status. Kaplan–Meier curves were constructed to compare the survival functions by health insurance status, with a log‐rank test used to test for differences in these survival functions. Cox proportional hazards regression models were used to assess the hazard ratios (HR) for the association between binary health insurance and cancer‐specific mortality. The proportional hazards assumption was assessed with a goodness‐of‐fit test [26] and graphically with plots of the Schoenfeld residuals [27]. If the assumption was not met, an extended Cox regression model would be used to allow for the time‐dependent covariate. We performed two‐sided tests at a significance level of α = 0.05.

To determine if health insurance is a mediator of the association between ADI and survival, a weighted mediation approach was performed to identify the natural direct effect, the natural indirect effect, the total effect, and the proportion mediated. This analysis was done using the mets package in R, which allows for time‐to‐event outcomes with Cox proportional hazards regression models [28]. Within the mets package, formula‐based asymptotic variance was used for standard errors. Traditionally, mediation analyses require several assumptions, including no unmeasured confounding of the exposure‐outcome, exposure‐mediator, and mediator‐outcome relationships conditional on the included variables (sequential ignorability), correct model specification, and no exposure‐mediator interaction [29]. To support the sequential ignorability association, confounders were identified a priori using our DAG based on a literature review and subject‐matter expertise, including measured and unmeasured variables. To test for exposure‐mediator interaction, we used an interaction term between ADI and health insurance status. No meaningful interaction was observed (*p* = 0.69), indicating that this assumption held.

### 2.4 Sensitivity Analyses

Because of known differences in survival by cancer type [30], it was determined a priori to stratify the survival models by the three main cancer groups (hematologic malignancies, CNS tumors, and extracranial solid tumors) [31, 32]. Additionally, because the standard packages for causal mediation analysis in R do not allow for a non‐ordinal multi‐category mediator with a time‐to‐event outcome, we were limited to binary categorizations of health insurance. In supplemental analyses, we also considered other binary categorizations of insurance (public vs. non‐public [including private, insured NOS, or uninsured], insured NOS vs. non‐insured NOS [including private, public, or uninsured], and uninsured vs. insured [including public, private, or insured NOS]) as mediator variables.

## 3 Results

After the removal of individuals with missing data on health insurance (*n* = 1190, 17.04% of eligible sample), a total of 5792 children were included in the study (Figure S2); 2069 were diagnosed in Iowa and 3723 were diagnosed in Louisiana (Table 1). The median age at diagnosis was 10.00 years (IQR: 3.50–16.50). The median year of diagnosis differed by state; it was 2013 (IQR: 2010–2016) in Iowa and 2011 (IQR: 2006–2016) in Louisiana (*p* < 0.0001). Overall, 53.71% of the children diagnosed with cancer were male. The racial/ethnic distribution differed by state (*p* < 0.0001). Among those diagnosed in Iowa 83.62% of the children were non‐Hispanic White and 9.09% were Hispanic. Among those diagnosed in Louisiana, 60.84% of children were non‐Hispanic White, and 32.07% of the children were non‐Hispanic Black. There was no difference in the distribution of cancer groups by state (*p* = 0.29); the most common cancer group was extracranial solid tumors (40.99%), followed by hematologic malignancies (40.78%) and CNS tumors (18.23%). There was a greater proportion of children diagnosed in rural census tracts in Iowa compared with Louisiana (40.16% vs. 15.71%, *p* < 0.0001). A greater proportion of children diagnosed in Iowa were in the most deprived neighborhoods (Q4) than in Louisiana (28.08% vs. 22.88%, *p* < 0.0001).

**TABLE 1:** Demographic characteristics of included study participants, children aged 0–19 at cancer diagnosis from 2000 to 2020 in Iowa and Louisiana, *n* = 5792.

	Total, *N* = 5792	Iowa, *N* = 2069	Louisiana, *N* = 3723	*p* value
Age at diagnosis, median (IQR)	10.00 (3.50–16.50)	10.00 (3.50–16.50)	10.00 (3.50–16.50)	0.42
Year of diagnosis, median (IQR)	2012 (2008–2016)	2013 (2010–2016)	2011 (2006–2016)	< 0.0001
Sex				0.54
Male	3111 (53.71%)	1123 (54.28%)	1988 (53.40%)	
Female	2681 (46.29%)	946 (45.72%)	1735 (46.60%)	
Race/ethnicity				< 0.0001
NH White	3995 (68.97%)	1730 (83.62%)	2265 (60.84%)	
NH Black	1299 (22.43%)	105 (5.07%)	1194 (32.07%)	
NH other	118 (2.04%)	46 (2.22%)	72 (1.93%)	
Hispanic (any race)	380 (6.56%)	188 (9.09%)	192 (5.16%)	
Cancer groups				0.29
Extracranial solid tumors	2374 (40.99%)	873 (42.19%)	1501 (40.32%)	
Hematologic malignancies	2362 (40.78%)	817 (39.49%)	1545 (41.50%)	
CNS tumors	1056 (18.23%)	379 (18.32%)	677 (18.18%)	
Rurality				< 0.0001
Rural	1416 (24.45%)	831 (40.16%)	585 (15.71%)	
Urban	4376 (75.55%)	1238 (59.84%)	3138 (84.29%)	
Area Deprivation Index				< 0.0001
Q1 (least deprived)	726 (12.53%)	100 (4.83%)	626 (16.81%)	
Q2	1469 (25.36%)	605 (29.24%)	864 (23.21%)	
Q3	2164 (37.36%)	783 (37.84%)	1381 (37.09%)	
Q4 (most deprived)	1433 (24.74%)	581 (28.08%)	852 (22.88%)	
Health insurance status				< 0.0001
Private	2439 (42.11%)	1182 (57.13%)	1257 (33.76%)	
Public	2402 (41.47%)	661 (31.59%)	1741 (46.76%)	
Insured NOS	795 (13.73%)	187 (9.04%)	608 (16.33%)	
Uninsured	156 (2.69%)	39 (1.88%)	117 (3.14%)	
ACA Status				< 0.0001
Pre‐ACA	3830 (66.13%)	1035 (50.02%)	2795 (75.07%)	
Post‐ACA	1962 (33.87%)	1034 (49.98%)	928 (24.93%)	

Abbreviations: A/PI, Asian/Pacific Islander; AI/AN, American Indian/Alaskan Native; CNS, central nervous system; IQR, interquartile range; NH, non‐Hispanic; NOS, not otherwise specified.

The distribution of health insurance also varied by state (*p* < 0.0001). Compared with children in Iowa, a greater proportion of children in Louisiana had public insurance (46.76% vs. 31.59%) or were uninsured (3.14% vs. 1.88%). Due to the exclusion of the majority of cases before 2007 in Iowa and the later start of ACA implementation in Louisiana, a greater proportion of cases in Iowa were diagnosed post‐ACA implementation (49.98% vs. 24.93%, *p* < 0.0001). Before the implementation of the ACA, 38.83% of children had private insurance (both states combined), 41.12% had public insurance, 16.87% were insured NOS, and 3.19% were uninsured (Table S1). Post‐ACA implementation, 48.52% of children had private insurance, 42.15% had public insurance, 7.59% were insured NOS, and 1.17% were uninsured (both states combined).

Table 2 shows the association between ADI, other covariates, and health insurance status. In analyses also adjusted for sex, race/ethnicity, cancer group, age, rurality, year of diagnosis, and state, compared with children in the least deprived neighborhoods, children in the most deprived neighborhoods were more likely to have non‐private insurance (Q3 vs. Q1: aPR = 1.36, 95% CI = 1.20–1.55; Q4 vs. Q1: aPR = 1.52, 95% CI = 1.33–1.74). Compared with non‐Hispanic White children, non‐Hispanic Black children were 40% more likely to have non‐private insurance (aPR = 1.40, 95% CI = 1.29–1.52), and Hispanic children were 51% more likely to have non‐private insurance (aPR = 1.51, 95% CI = 1.33–1.71). The odds of having non‐private insurance decreased with increasing age (*p* < 0.0001) and year of diagnosis (*p* = 0.0003), and the odds of having non‐private insurance were higher for children in Louisiana vs. Iowa (*p* < 0.0001).

**TABLE 2:** Association between ADI, other covariates, and health insurance status (non‐private vs. private) among children aged 0–19 at cancer diagnosis from 2000 to 2020 in Iowa and Louisiana, *n* = 5792.

	PR (95% CI)	*p* value	aPR* (95% CI)	*p* value
Area Deprivation Index		< 0.0001		< 0.0001
Q1 (least deprived)	Reference		Reference	
Q2	1.03 (0.94‐1.14)		1.11 (0.98‐1.28)	
Q3	1.34 (1.22‐1.46)		1.36 (1.20‐1.55)	
Q4 (most deprived)	1.56 (1.43‐1.70)		1.52 (1.33‐1.74)	
Sex		0.11		0.30
Male	Reference		Reference	
Female	0.96 (0.92‐1.01)		0.96 (0.90‐1.03)	
Race/ethnicity		< 0.0001		< 0.0001
NH White	Reference		Reference	
NH Black	1.71 (1.64‐1.78)		1.40 (1.29‐1.52)	
NH other	1.03 (0.86‐1.24)		1.03 (0.80‐1.34)	
Hispanic (any race)	1.56 (1.46‐1.67)		1.51 (1.33‐1.71)	
Cancer groups		0.24		0.51
Hematologic malignancies	Reference		Reference	
CNS tumors	1.00 (0.94‐1.06)		0.99 (0.90‐1.08)	
Extracranial solid tumors	0.96 (0.91‐1.01)		0.97 (0.90‐1.05)	
Age at diagnosis	0.99 (0.98‐0.99)	< 0.0001	0.99 (0.98‐0.99)	< 0.0001
Rurality		0.21		0.95
Urban	Reference		Reference	
Rural	0.96 (0.91‐1.01)		1.00 (0.91‐1.09)	
Year of diagnosis	0.98 (0.98‐0.98)	< 0.0001	0.99 (0.98‐0.99)	0.0003
State		< 0.0001		< 0.0001
Iowa	Reference		Reference	
Louisiana	1.55 (1.46‐1.63)		1.41 (1.30‐2.54)	

Abbreviations: aPR, adjusted prevalence ratio; CNS, central nervous system; NH, non‐Hispanic; PR, prevalence ratio.

* Adjusted for all variables in the table, including Area Deprivation Index, sex, race/ethnicity, cancer groups, age at diagnosis, rurality, year of diagnosis, state.

**FIGURE 1:**
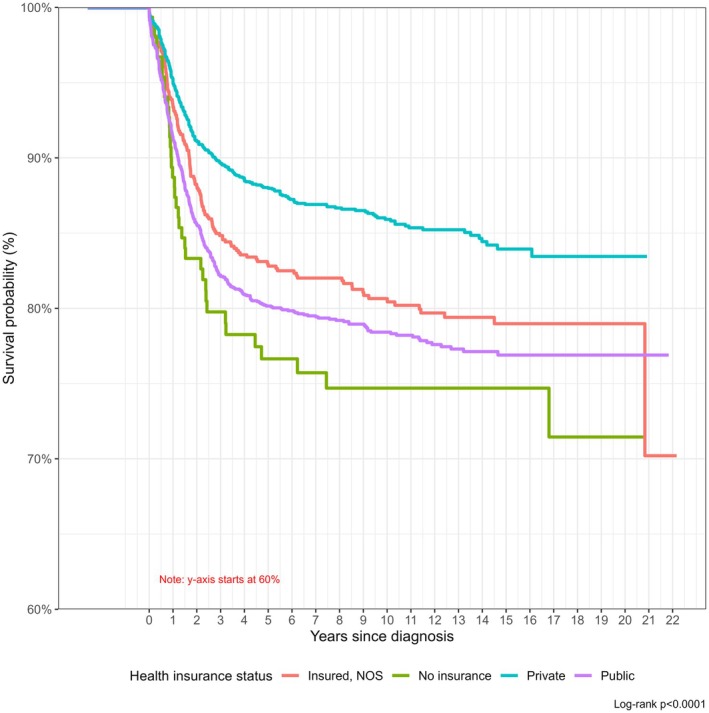
Kaplan–Meier curve showing differences in survival probability over time by health insurance status among children aged 0–19 diagnosed with cancer in Iowa and Louisiana from 2000 to 2020, *n* = 5792 with log‐rank test of survival differences (*p* < 0.0001).

Health insurance status was associated with pediatric cancer survival (log‐rank *p* value < 0.0001) before adjusting for covariates (Figure 1). Survival probability over time was highest among those with private insurance, followed by those who were insured NOS, followed by those with public insurance, and those who were uninsured at diagnosis. Children who died from causes unrelated to cancer were censored at the time of their death; these cases represented approximately 11% of all deaths. The proportional hazards assumption was met for all analyses. In adjusted models, compared with children with private insurance, the hazard of cancer death was higher among children without private insurance (aHR = 1.32, 95% CI = 1.13–1.55) (Table 3). Among the other covariates, sex, race/ethnicity, cancer type, and ADI were also associated with survival. Compared with children in the least deprived neighborhoods, those in the most deprived neighborhoods have a 48% higher hazard of cancer death (aHR = 1.48, 95% CI: 1.14–1.91). After stratifying by cancer type, the association between health insurance status and survival was only statistically significant among children with hematologic malignancies (aHR = 1.33, 95% CI: 1.01–1.75), and this point estimate was similar to the overall point estimate (Table S2).

**TABLE 3:** Association between health insurance status and survival among children aged 0–19 at cancer diagnosis from 2000 to 2020 in Iowa and Louisiana, *n* = 5792.

	HR (95% CI)	*p* value	aHR* (95% CI)	*p* value
Health insurance status		< 0.0001		0.0002
Private	Reference		Reference	
Non‐private	1.59 (1.39‐1.82)		1.32 (1.13‐1.55)	
Area Deprivation Index		< 0.0001		0.03
Q1 (least deprived)	Reference		Reference	
Q2	1.23 (0.98‐1.56)		1.25 (0.97‐1.60)	
Q3	1.28 (1.03‐1.60)		1.22 (0.96‐1.56)	
Q4 (most deprived)	1.63 (1.30‐2.03)		1.48 (1.14‐1.91)	
Sex		< 0.0001		0.001
Male	Reference		Reference	
Female	0.79 (0.70‐0.89)		0.81 (0.71‐0.92)	
Race/ethnicity		< 0.0001		< 0.0001
NH White	Reference		Reference	
NH Black	1.72 (1.51‐1.96)		1.50 (1.29‐1.76)	
NH other	1.58 (1.10‐2.29)		1.71 (1.15‐2.55)	
Hispanic (any race)	0.92 (0.70‐1.20)		0.85 (0.62‐1.16)	
Cancer groups		< 0.0001		< 0.0001
Hematologic malignancies	Reference		Reference	
CNS tumors	2.76 (2.38‐3.20)		2.89 (2.46‐3.41)	
Extracranial solid tumors	1.37 (1.19‐1.60)		1.44 (1.23‐1.68)	
Age at diagnosis	1.00 (0.99‐1.00)	0.32	1.00 (0.99‐1.01)	0.69
Rurality		0.91		0.71
Urban	Reference		Reference	
Rural	1.01 (0.89‐1.14)		1.03 (0.88‐1.21)	
Year of diagnosis	0.98 (0.97‐0.99)	< 0.0001	0.99 (0.98‐1.00)	0.09
State		0.0003		0.24
Iowa	Reference		Reference	
Louisiana	1.24 (1.11‐1.40)		1.10 (0.94‐1.29)	

Abbreviations: aHR, adjusted hazard ratio; HR, hazard ratio; NOS, not otherwise specified.

* Adjusted for all variables in the table, including health insurance status, Area Deprivation Index, sex, race/ethnicity, cancer groups, age at diagnosis, rurality, year of diagnosis, and state.

Health insurance status (non‐private vs. private) was a mediator of the association between ADI and survival (natural indirect effect Q2: aHR = 1.02, 95% CI: 1.00–1.03; Q3: aHR = 1.05, 95% CI: 1.02–1.07; Q4: aHR = 1.07, 95% CI: 1.03–1.11) (Table 4). Compared with those in the least deprived neighborhoods (Q1), the proportion of the effect of ADI on survival mediated through health insurance status (non‐private vs. private) was 7.33%, 17.57%, and 14.59% for Q2, Q3, and Q4 vs. Q1, respectively. The presence of health insurance (uninsured vs. insured) was not a mediator of the association between ADI and survival (% mediated = 0.00 for all ADI levels) (Table S3). Compared with those in the least deprived neighborhoods (Q1), the proportion of the effect of ADI on survival mediated through health insurance status (non‐public vs. public) was 3.79%, 7.33%, and 6.37% for Q2, Q3, and Q4 vs. Q1, respectively.

**TABLE 4:** Mediation analysis of health insurance status (non‐private vs. private) in the association between ADI and survival among children aged 0–19 at cancer diagnosis from 2000 to 2020 in Iowa and Louisiana, *n* = 5792.

	NDE	NIE	TE	% Mediated
Area Deprivation Index				
Q1 (least deprived)	Reference	Reference	Reference	Reference
Q2	1.28 (1.00–1.65)	1.02 (1.00–1.03)	1.31 (1.00–1.70)	7.33%
Q3	1.26 (0.99–1.61)	1.05 (1.02–1.07)	1.32 (1.01–2.82)	17.57%
Q4 (most deprived)	1.49 (1.15–1.93)	1.07 (1.03–1.11)	1.59 (1.18–2.14)	14.59%

Abbreviations: NDE, natural direct effect; NIE, natural indirect effect; TE, total effect.

## 4 Discussion

In this population‐based study, we observed that among children with cancer in Iowa and Louisiana, children in more deprived neighborhoods and racial/ethnic minorities were more likely not to be privately insured. Additionally, we also observed insurance‐, ADI‐, and racial/ethnic‐based disparities in survival. While individual‐level health insurance status was a mediator of neighborhood‐level disparities in pediatric cancer survival, it did not explain a large proportion of the observed disparities and thus is not the primary driver, with over 80% of neighborhood‐level disparities acting through other mechanisms. This suggests that structural and systemic SDOH, beyond just individual insurance coverage, may play a significant role in shaping neighborhood‐level pediatric cancer outcomes and may require targeted, upstream interventions to meaningfully reduce disparities. These results may contribute to the growing literature of multilevel barriers in pediatric cancer care across the cancer‐control continuum and highlight the importance of exploring broader policy and system‐level interventions to reduce disparities.

Insurance coverage among children with cancer has improved post‐ACA implementation, yet gaps remain, underscoring the need to examine structural determinants of access to care. In our study, we observed that 2.69% of children with cancer were uninsured at the time of diagnosis with a higher proportion of uninsured children in Louisiana than Iowa. Comparatively, it was estimated that about 5% of all children 18 and under in the United States were uninsured in 2023, and 41% covered by public insurance [33]. In the post ACA implementation era, only 1.17% of children in our study were uninsured, reflecting general improvements in insurance rates among children seen nationally [34]. The results are also consistent with an analysis of the National Cancer Database that reported greater improvements in insurance coverage post‐ACA implementation among racial/ethnic minorities and children with cancer in low‐income zip codes [35]. Despite the improvements in insurance rates, the persistence of uninsurance, especially in vulnerable communities, may exacerbate barriers to timely referral and access to care. Given that most insurance in the US is employer‐based, parental employment status may also play a critical role in access to care and overall outcomes, warranting further investigation [36]. In addition to differences in health insurance status by state and year of diagnosis, we also observed that insurance status differed by ADI, race/ethnicity, and age at diagnosis. In a study of SEER data, health insurance status was a mediator of racial/ethnic disparities in pediatric cancer survival, reflecting complex, multifactorial pathways driving pediatric cancer disparities [37].

Our results are consistent with prior research demonstrating associations between insurance type and pediatric cancer survival, with those that are uninsured or covered by public insurance having a higher hazard of cancer death [14, 38]. Previous studies have shown that underinsurance, even when formal coverage is present, may limit access to timely or specialized care due to high deductibles, limited provider networks, and coverage exclusion [39, 40]. Although health insurance may not be seen as an easily modifiable exposure, our findings indicate that policy intervention, such as Medicaid expansion, continuity of coverage during treatment, and cost‐sharing protection, can functionally “modify” these factors. Provisions within the ACA have implications not only for children actively undergoing treatment for cancer, but also for childhood cancer survivors, such as coverage on parents' insurance up to the age of 26, no prohibition of discrimination on the basis of health status, and no annual or lifetime coverage limits [41].

This study is novel in its examination of whether individual‐level health insurance status is a mediator of the association between ADI and pediatric cancer survival. We observed that health insurance status accounted for less than 20% of the association between neighborhood deprivation and pediatric cancer survival. This suggests that while health insurance is one factor along the path between neighborhood deprivation and survival, other factors, perhaps even factors that are easier to modify, such as clinical trials education, or access to clinical trials and supportive care services, could account for the natural direct effect and may also be potential intervention targets to improve neighborhood‐level disparities. For example, it has been reported that non‐White children in the United States in the National Childhood Cancer Registry are less likely to be enrolled on a Children's Oncology Group study [42]. Beyond clinical trial participation, acuity at presentation may be another contributor to neighborhood‐level disparities if children in deprived neighborhoods experience delays in diagnosis. Acuity at presentation has previously been observed to be a mediator of Black/White disparities in early mortality among children with acute myeloid leukemia [43]. The integration of oncological social workers and patient navigation by cancer centers may help address the needs of pediatric cancer patients and their families [44, 45].

### 4.1 Policy Implications

From a policy perspective, our findings support multilevel approaches that target not only insurance coverage and access, but neighborhood‐level barriers to high‐quality oncologic and primary care, transportation burden, and delays in diagnosis and treatment. Policy efforts can be extended to incorporate investments in care coordination networks, telehealth infrastructure, and support for patient navigation and social work integration. These changes, in combination with efforts directed toward health insurance policy, such as Medicaid coverage expansion and continuity provisions, may improve equity in pediatric cancer outcomes.

### 4.2 Strengths and Limitations

Our study has several strengths, including its representative population‐based cohort; however, it does have several limitations. First, insurance was only measured at the time of diagnosis, so we were not able to assess any lapses or changes in insurance status, which are not uncommon [46]. Second, although the state cancer registries adhere to high data collection standards, they do not contain data on variables such as individual‐level income, parental employment, healthcare and clinical trial access, and out‐of‐pocket costs; as such, our study may be subject to residual confounding. Causal mediation analyses should be interpreted under the required assumptions. Although no formal tests were undertaken to examine sequential ignorability, previous work has shown that natural direct effects can be identified even in the presence of unmeasured exposure‐outcome confounding as long as there is no additive interaction between the mediator and the unmeasured confounder, indicating our methods should be robust to unmeasured confounding of this nature [47]. Third, we were limited to binary classifications of insurance for mediation analyses, which are not able to capture differences between public insurance, private insurance, and those that are uninsured. Quartile‐based categorizations of ADI also assume a stepwise effect; while a continuous variable may demonstrate the true dose–response relationship, the quartile method was chosen to improve interpretability of effect estimates, which can be interpreted as the weighted average of the effect in each quartile. Additionally, although the vast majority of deaths in this population were due to cancer, our results may be biased if non‐cancer deaths are informative. Finally, our results may only be generalizable to populations with similar sociodemographic characteristics.

## 5 Conclusion

While health insurance status is associated with pediatric cancer survival, our data suggest that individual‐level socioeconomic status only partially mediates the path between neighborhood deprivation and pediatric cancer survival. This suggests that while health insurance is an important driver of health, other mechanisms are likely to contribute to neighborhood‐level survival disparities in pediatric cancer. Policy changes targeting insurance continuity, expansion, and affordability remain vital, but these changes should be part of a broader strategy addressing multilevel aspects of the pediatric cancer care continuum.

## Author Contributions

**Emma Hymel:** conceptualization (equal), formal analysis (equal), funding acquisition (equal), methodology (equal), writing – original draft (equal), writing – review and editing (equal). **Cheng Zheng:** conceptualization (equal), methodology (equal), supervision (equal), writing – review and editing (equal). **Jenna Allison:** conceptualization (equal), methodology (equal), supervision (equal), writing – review and editing (equal). **Kendra L. Ratnapradipa:** conceptualization (equal), methodology (equal), supervision (equal), writing – review and editing (equal). **Edward S. Peters:** conceptualization (equal), methodology (equal), supervision (equal), writing – review and editing (equal). **Sarah H. Nash:** data curation (equal), methodology (equal), writing – review and editing (equal). **Mei‐Chin Hsieh:** data curation (equal), methodology (equal), writing – review and editing (equal). **Shinobu Watanabe‐Galloway:** conceptualization (equal), funding acquisition (equal), methodology (equal), project administration (equal), supervision (equal), writing – review and editing (equal).

## Funding

This work was supported by the Child Health Research Institute, University of Nebraska Medical Center.

## Ethics Statement

The study was approved by the Institutional Review Board at the University of Nebraska Medical Center (0367–23‐EX). Informed consent was not required because the study used population‐based cancer registry data collected for public health surveillance purposes.

## Conflicts of Interest

The authors declare no conflicts of interest.

## Supporting information


**Table S1:** Health insurance status pre/post Affordable Care Act implementation among children aged 0–19 at cancer diagnosis from 2000 to 2020 in Iowa and Louisiana.
**Table S2:** Association between health insurance and survival stratified by cancer type among children aged 0–19 diagnosed with cancer in Iowa and Louisiana from 2000 to 2020, *n* = 5792.
**Table S3:** Mediation analysis of alternative categorizations of health insurance status in the association between ADI and survival among children aged 0–19 at cancer diagnosis from 2000 to 2020 in Iowa and Louisiana, *n* = 5792.
**Figure S1:** Directed acyclic graph used to identify variables to adjust for in the multivariable analysis.
**Figure S2:** Flowchart of included study participants.

## Data Availability

The data supporting this study are not publicly available due to ethical and privacy concerns. Access to the data must be granted from the Iowa Cancer Registry and Louisiana Tumor Registry.
