# Supplementary material for: Health Insurance as a Mediator of Neighborhood Deprivation and Pediatric Cancer Survival: An Analysis of State Cancer Registry Data
Source: Cancer Med. 2026 Mar 27;15(4):e71755. doi: 10.1002/cam4.71755 (PMC13140461; doi:10.1002/cam4.71755)
Supplement: Supplementary file 1 — Table S1: Health insurance status pre/post Affordable Care Act implementation among children aged 0–19 at cancer diagnosis from 2000 to 2020 in Iowa and Louisiana. Table S2: Association between health insurance and survival stratified by cancer type among children aged 0–19 diagnosed with cancer in Iowa and Louisiana from 2000 to 2020, n = 5792. Table S3: Mediation analysis of alternative categorizations of health insurance status in the association between ADI and survival among children aged 0–19 at cancer diagnosis from 2000 to 2020 in Iowa and Louisiana, n = 5792. Figure S1: Directed acyclic graph used to identify variables to adjust for in the multivariable analysis. Figure S2: Flowchart of included study participants. [file CAM4-15-e71755-s001.docx]

Supplemental Table 1. Health insurance status pre/post Affordable Care Act implementation among children aged 0-19 at cancer diagnosis from 2000-2020 in Iowa and Louisiana

|  | Pre-ACA, N= 3,830 | Post-ACA, N= 1,962 |
| --- | --- | --- |
| Health insurance status  Private  Public  Insured NOS  Uninsured | 1,487 (38.83%)  1,575 (41.12%)  646 (16.87%)  122 (3.19%) | 952 (48.52%)  827 (42.15%)  149 (7.59%)  34 (1.17%) |

Abbreviations: ACA, Affordable Care Act; NOS, not otherwise specified

Supplemental Table 2. Association between health insurance and survival stratified by cancer type among children aged 0-19 diagnosed with cancer in Iowa and Louisiana from 2000-2020, n=5,792

|  | aHR (95% CI) ^a^ | p-value |
| --- | --- | --- |
| **Hematologic malignancies**  Health insurance status  Private  Non-Private | Reference  1.33 (1.01-1.75) | 0.04 |
| **CNS tumors**  Health insurance status  Private  Non-Private | Reference  1.25 (0.96-1.61) | 0.10 |
| **Extracranial solid tumors**  Health insurance status  Private  Non-Private | Reference  1.38 (0.93-2.04) | 0.11 |

^a^Adjusted for: ADI, state, rurality, sex, race/ethnicity, age at diagnosis, and year of diagnosis

Abbreviations: CNS, central nervous system; aHR, adjusted hazard ratio

Supplemental Table 3. Mediation analysis of alternative categorizations of health insurance status in the association between ADI and survival among children aged 0-19 at cancer diagnosis from 2000-2020 in Iowa and Louisiana, n=5,792

|  | NDE | NIE | TE | % Mediated |
| --- | --- | --- | --- | --- |
| Public vs. Non-Public |  |  |  |  |
| Area Deprivation Index  Q1 (least deprived)  Q2  Q3  Q4 (most deprived) | Reference  1.29 (1.00-1.66)  1.28 (1.00-1.64)  1.54 (1.19-2.01) | Reference   - 1. (1.00-1.05)   1.02 (0.99-1.07)  1.03 (1.00-1.07) | Reference  1.30 (1.00-1.74)  1.31 (0.99-1.75)  1.59 (1.19-2.15) | Reference  3.79%  7.33%  6.37% |
| Insured NOS vs. Non-Insured NOS |  |  |  |  |
| Area Deprivation Index  Q1 (least deprived)  Q2  Q3  Q4 (most deprived) | Reference  1.25 (0.98-1.61)  1.28 (1.01-1.63)  1.57 (1.22-2.02) | Reference  1.00 (1.00-1.00)  1.00 (0.99-1.00)  1.00 (0.99-1.00) | Reference  1.25 (0.98-1.61)  1.18 (1.00-1.63)  1.57 (1.21-2.02) | Reference  0.00%  0.00%  0.00% |
| Uninsured vs. Insured |  |  |  |  |
| Area Deprivation Index  Q1 (least deprived)  Q2  Q3  Q4 (most deprived) | Reference  1.25 (0.98-1.61)  1.28 (1.01-1.62)  1.57 (1.22-2.01) | Reference  1.00 (0.99-1.01)  1.00 (0.99-1.00)  1.00 (0.99-1.01) | Reference  1.25 (0.97-1.63)  1.28 (1.00-1.62)  1.57 (1.21-2.03) | Reference  0.00%  0.00%  0.00% |

Abbreviations: NDE, natural direct effect; NIE, natural indirect effect; TE, total effect; NOS, not otherwise specified

Non-Public included Private Insurance, Insured NOS, and Uninsured. Non-Insured NOS included Private Insurance, Public Insurance, and Uninsured.

Supplemental Figure 1. Directed acyclic graph used to identify variables to adjust for in the multivariable analysis


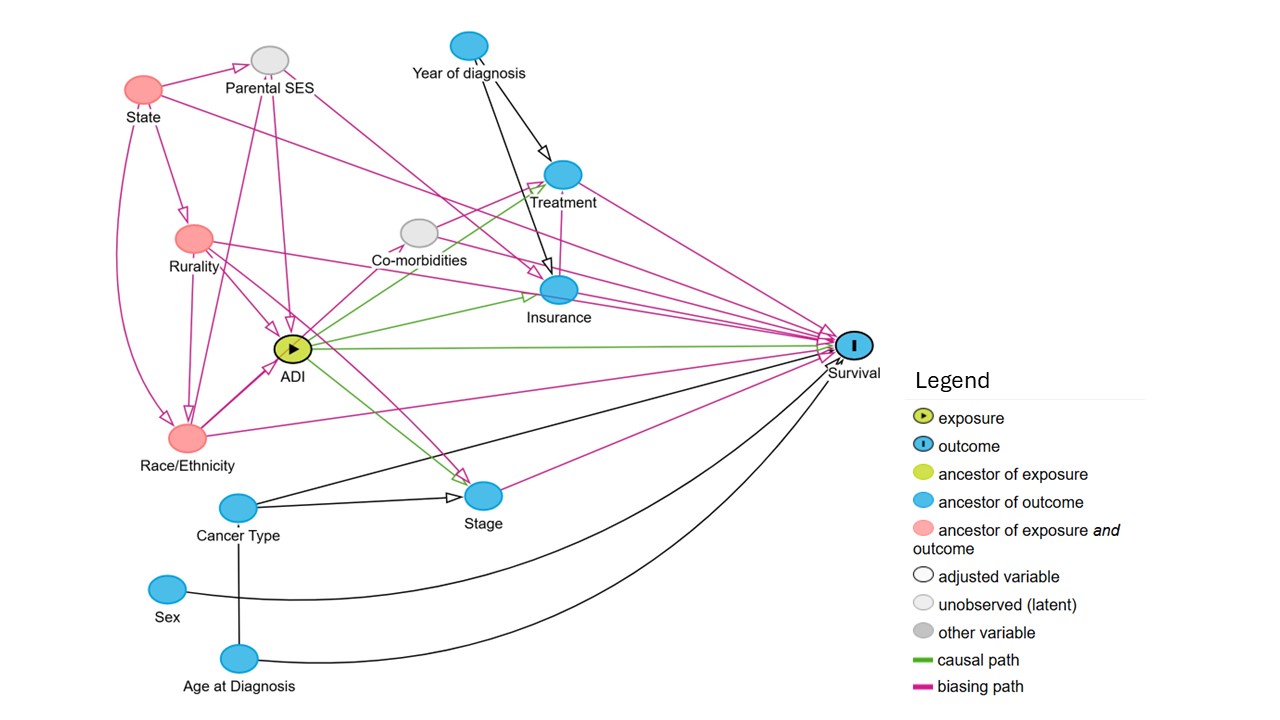


Supplemental Figure 2. Flowchart of included study participants

**
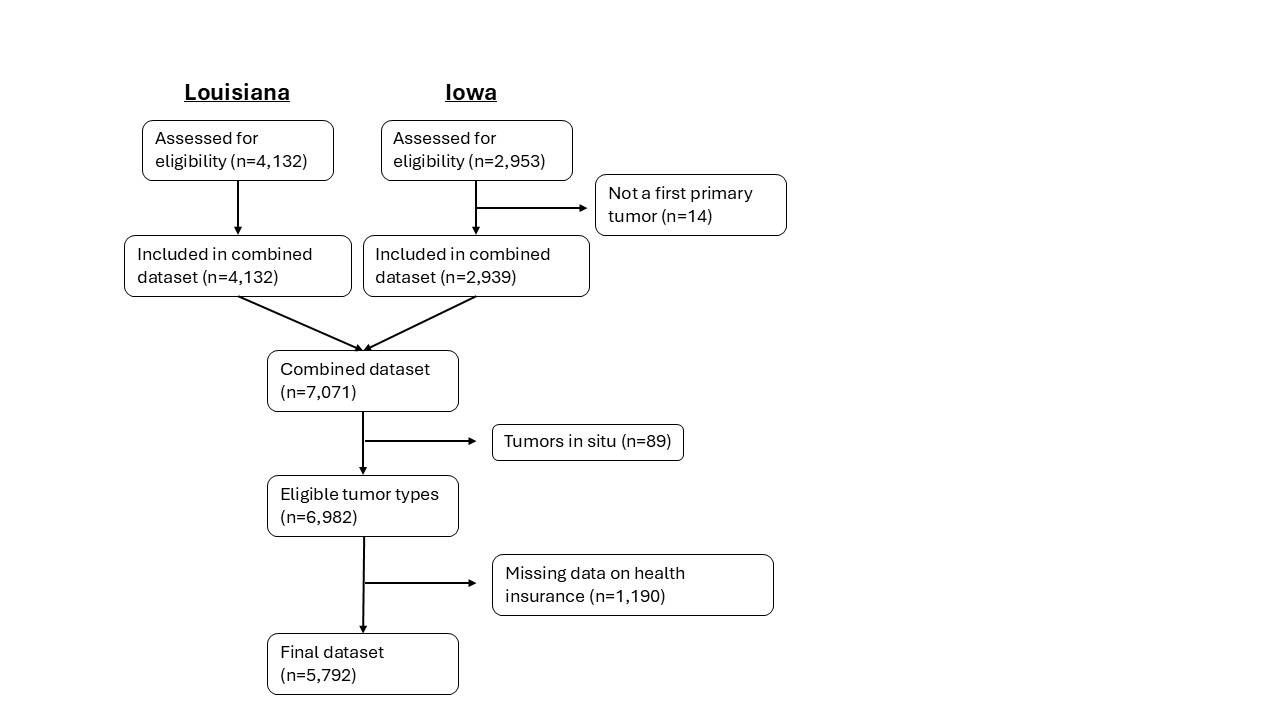
**
